# Review of Structural Modification and Development of Novel Tramadol Derivatives

**DOI:** 10.3390/molecules31071177

**Published:** 2026-04-02

**Authors:** Ni Wang, Xiaoli Zhou, Jingwen Wang, Lixin Sun, Bo Liu, Lihui Yin

**Affiliations:** 1 School of Pharmacy, Shenyang Pharmaceutical University, Shenyang 110016, China; wangni0215@foxmail.com; 2 National Institutes for Food and Drug Control, Beijing 102629, China; zhouxiaoli@nifdc.org.cn (X.Z.); wangjingwen@nifdc.org.cn (J.W.); 3 State Key Laboratory of Drug Regulatory Science, Beijing 102629, China

**Keywords:** tramadol, analgesics, structure–activity relationship, opioid receptors

## Abstract

Tramadol acts via μ-opioid receptor agonism and monoamine reuptake inhibition but is clinically limited by metabolic dependence, interindividual variability, and addiction risks. Structural modification aims to resolve these limitations. This review systematically summarizes tramadol’s structure–activity relationships and mechanisms, focusing on key strategies for structural optimization. Major advances include: (i) synergistic strategies, such as tramadol–celecoxib cocrystals (tramadol and celecoxib coexist in the supramolecular crystal network at a 1:1 molar ratio), achieving multimodal analgesia at lower doses; (ii) mechanism-balancing strategies such as tapentadol (derivatives of tramadol with a dual mechanism of action), which enhance μ-opioid agonism and norepinephrine reuptake inhibition while attenuating serotonergic effects to improve efficacy; (iii) metabolic optimization utilizing M1 analogues to circumvent CYP2D6 polymorphisms (tramadol is metabolized by this enzyme into the active metabolite M1 to exert analgesic effects); and (iv) pharmacophore optimization leveraging tramadol–morphine homology and “message–address” concepts to design selective ligands. Novel derivatives demonstrate improved potency and metabolic stability but continue to face challenges regarding opioid risks and clinical translation. Future research should integrate rational drug design, delivery systems, and personalized medicine to facilitate the development of safer next-generation analgesics.

## 1. Introduction

Tramadol is a widely utilized analgesic primarily indicated for the treatment of moderate to severe pain, including acute traumatic pain, renal colic, biliary colic, and labor pain [1]. The compound was first synthesized in 1962 by Grünenthal GmbH in Germany and was introduced to the market as tramadol in 1977. It subsequently entered the United States market in 1995 [2].

Owing to its distinctive pharmacological advantages, tramadol has sustained high market demand. In addition to several reference products such as Ultram (immediate-release tablets), ConZip (extended-release capsules), and Ultram ER (extended-release tablets), the market contains more than 160 generic formulations produced by over 100 pharmaceutical manufacturers [3]. It is available via various routes of administration, including oral drops, capsules, immediate- and extended-release tablets, suppositories, and injectable solutions for intramuscular, intravenous, and subcutaneous administration [1].

In clinical practice, tramadol is utilized across diverse therapeutic settings and is particularly vital in postoperative pain management. Its co-administration with non-opioid analgesics, such as acetaminophen, is consistent with principles of pharmacological synergy and aligns with recommended multimodal analgesia strategies for postoperative pain control [4]. Although the analgesic potency of tramadol is approximately one-tenth that of morphine [5], it exhibits a more favorable safety profile. Compared with morphine and other opioid analgesics, tramadol is associated with a significantly lower risk of respiratory depression [6].

Beyond its analgesic properties, tramadol demonstrates immunomodulatory and anti-inflammatory effects, which support its use in varied pain management scenarios. Regarding immunomodulation, tramadol exerts immunostimulatory activity. Studies in rodent models treated with tramadol and in cells isolated from patients indicate that its mechanisms include enhancement of natural killer (NK) cell activity, promotion of splenocyte proliferation [7], and augmentation of phagocytic activity in phagocytes [8]. Clinical studies provide further evidence. In patients with gastric cancer receiving tramadol-based patient-controlled analgesia, the reduction in NK cell counts was significantly less pronounced than in patients treated with morphine [9]. Tramadol also appears to contribute to the maintenance of immune homeostasis. In vitro studies have shown that morphine reduces the phagocytic function of monocytes [10] and that morphine, methadone, and oxycodone all suppress interleukin-2 (IL-2)-stimulated interleukin-6 (IL-6) production by peripheral blood mononuclear cells, whereas tramadol does not exert such inhibitory effects [11]. These findings indicate that tramadol interferes less with cytokine secretion by immune cells, making it more suitable for patients requiring long-term analgesia while preserving immune function.

Evidence further suggests that tramadol and its μ-opioid receptor (MOR)-active metabolite, O-desmethyltramadol, produce significantly weaker immunosuppressive effects than morphine [12]. Regarding anti-inflammatory activity, tramadol attenuates inflammatory responses in both rat models of in vivo inflammation [13] and mouse models of venous thrombosis [14]. In vitro, tramadol more effectively inhibits lipopolysaccharide (LPS)-induced release of tumor necrosis factor (TNF) and IL-8 from U-937 cells compared with morphine [15]. Clinical studies demonstrate that, in pediatric patients undergoing abdominal surgery, tramadol-based analgesia reduces levels of inflammatory markers, including IL-6, cortisol, and C-reactive protein (CRP), thereby helping to alleviate postoperative systemic inflammatory responses [16]. Due to the synergistic pharmacological actions encompassing analgesia, immunomodulation, and anti-inflammatory effects, clinicians widely prescribe tramadol for the management of acute and chronic pain, dental pain, cancer-related pain, and labor analgesia [17].

However, significant limitations remain in the clinical use of tramadol. First, because its mechanism of action involves multiple signaling pathways and its metabolism overlaps with those of various concurrently administered drugs, tramadol is highly susceptible to drug–drug interactions [18]. Therefore, patients receiving tramadol require careful monitoring to minimize the risk of potential adverse effects. Second, the adverse reactions associated with tramadol monotherapy warrant consideration. Overdose may result in drowsiness, headache, respiratory distress, bradycardia, coma, and death. Long-term use carries a risk of dependence, and drug discontinuation may lead to both typical and atypical withdrawal symptoms, most commonly including gastrointestinal cramping, bone pain, diarrhea, nausea, anxiety, and rhinorrhea [19,20]. Some withdrawal manifestations resemble those observed with selective serotonin reuptake inhibitors and may include atypical symptoms such as panic attacks, confusion, delusions, paranoid ideation, and severe anxiety [19,20]. Furthermore, some studies suggest that tramadol use may be associated with an increased risk of suicide [17].

To address these limitations and to improve both analgesic efficacy and safety, extensive efforts have focused on the structural optimization and modification of the tramadol molecule. For example, novel derivatives constructed by linking an N-methyl group to the cyclohexane ring do not bind to the MOR, yet they exhibit antinociceptive effects in both rat and mouse pain models [21]. Such structural modification strategies provide insights for the development of novel analgesics with enhanced efficacy, reduced abuse potential, and more favorable pharmacokinetic properties. In recent years, the field of tramadol structural modification and derivative development has achieved significant progress, establishing a framework for innovation in analgesic drug discovery. Therefore, this review systematically summarizes recent strategies for tramadol structural modification and the development of novel derivatives to provide guidance for the future development of next-generation analgesic agents.

## 2. Structure–Activity Relationships and Mechanisms of Action of Tramadol

### 2.1. Structure–Activity Relationships of Tramadol

**Figure 1:**
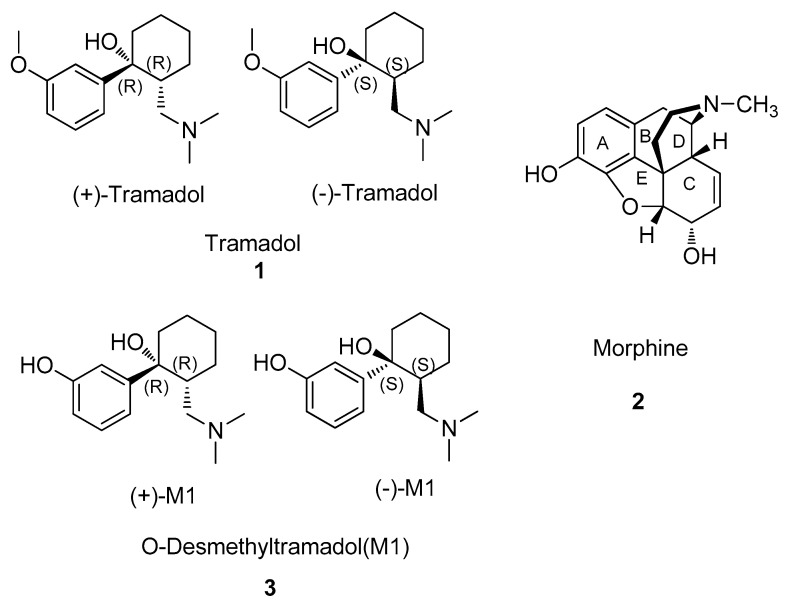
Structures of tramadol (**1**), morphine (**2**), and M1 (**3**).

Tramadol (**1**) was developed through the structural simplification and targeted modification of the classic phenolic opioid morphine (**2**), as shown in Figure 1. Its core structure consists of three primary components: a phenyl ring, a cyclohexane ring (with the hydroxyl group in the *trans* configuration, which is requisite for biological activity), and an aminomethyl side chain, in which the dimethylamino group serves as a critical pharmacophore. The structure–activity relationships (SARs) of tramadol are characterized by several key features.

First, the phenyl ring and the 3-methoxy substituent serve as essential pharmacophores for binding to the MOR. These structural features share chemical similarities with the A and C rings of the opioid analgesic morphine [22,23]. In terms of three-dimensional spatial orientation and electronic properties, the 3-methoxyphenyl moiety of tramadol effectively mimics the aromatic binding plane and polar interaction sites provided by the A ring of morphine. This pharmacophore mimicry allows tramadol to emulate the spatial conformation of endogenous opioid peptides and interact with corresponding binding sites on opioid receptors [24].

Second, the dimethylaminomethyl side chain is vital for inhibiting monoamine neurotransmitter reuptake, particularly that of serotonin (5-hydroxytryptamine, 5-HT). The dimethylamino group (-N(CH_3_)_2_) is the optimal substituent to maintain monoamine reuptake inhibitory activity; modification of its alkyl substituents (e.g., replacement with methylethyl or diethyl groups) reduces activity. The length of this side chain, consisting of a single methylene unit, is also optimal for pharmacological activity [24].

Finally, the cyclohexanol moiety provides a rigid scaffold that controls the stereochemical arrangement of the substituents. The relative configuration is critical for pharmacological activity. Tramadol is administered clinically as the (±)-cis isomeric mixture [25], where the dimethylaminomethyl side chain and the hydroxyl group are positioned in a cis orientation on the cyclohexane ring. This stereochemical arrangement favors an appropriate three-dimensional conformation for interaction with the MOR and monoaminergic targets. The hydroxyl group (-OH) participates in hydrogen bonding with the opioid receptor, thereby enhancing binding affinity and selectivity. Dehydroxylation leads to a substantial loss of activity [26]. Based on these SAR data, structural modifications of tramadol have been pursued to develop analogues with improved pharmacological profiles.

### 2.2. Mechanisms of Action of Tramadol

Tramadol, chemically designated 2-[(dimethylamino)methyl]-1-(3-methoxyphenyl) cyclohexanol, is a synthetic opioid analgesic classified as a 4-phenylpiperidine derivative. Commercially available tramadol is a racemic mixture of the (*R*,*R*)- and (*S*,*S*)-enantiomers and is utilized clinically primarily as a hydrochloride salt [27], which is readily soluble in water and ethanol. The dimethylaminomethyl moiety of tramadol is functionally homologous to the methylated cyclic nitrogen atoms in codeine and morphine and represents a key pharmacophoric element responsible for MOR binding [24].

#### 2.2.1. Metabolic Activation Mechanism

**Figure 2:**
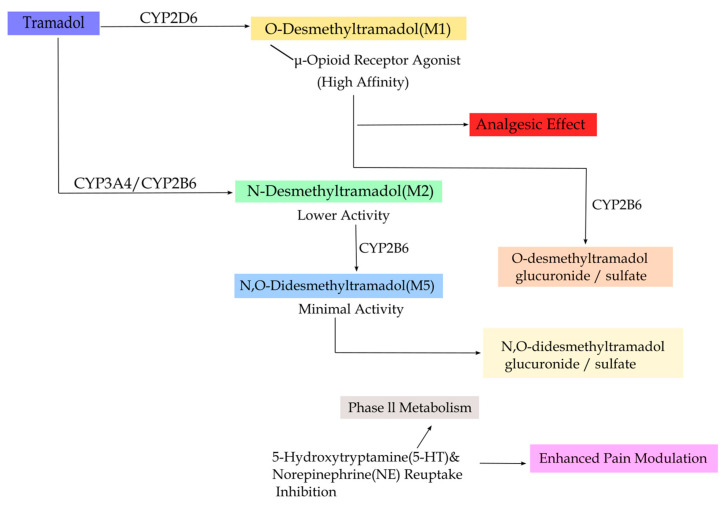
Metabolic activation pathway of tramadol.

Tramadol undergoes extensive metabolism via the cytochrome P450 (CYP) enzyme system [28]. O-demethylation catalyzed by CYP2D6 produces the active metabolite O-desmethyltramadol (M1, **3**, Figure 1), whereas N-demethylation mediated by CYP3A4 and CYP2B6 yields the metabolite M2, which can be further converted into downstream metabolites such as M5. Metabolites including M1 and M5 subsequently undergo conjugation reactions, such as glucuronidation or sulfation, to form water-soluble conjugates for renal excretion [29], as illustrated in Figure 2. The predominant metabolic pathway of tramadol is the formation of M1 catalyzed by the highly polymorphic CYP2D6 via O-demethylation [30,31]. Both in vitro and clinical studies have demonstrated that the parent compound, tramadol, is only a weak MOR agonist, whereas its metabolite M1 exhibits markedly higher affinity for the MOR—approximately 200–400-fold greater than the parent drug—and correspondingly more potent analgesic effects [12,32]. Consequently, M1 is the principal contributor to the analgesic activity of tramadol [32,33].

#### 2.2.2. Enantiomeric Synergism

Tramadol consists of two enantiomers that, alongside the active metabolite M1, contribute to overall analgesia through distinct, complementary mechanisms. Specifically, (−)-tramadol and its metabolite (−)-M1 primarily inhibit norepinephrine reuptake [32,34], whereas the (+)-tramadol enantiomer inhibits serotonin (5-HT) uptake via the serotonin transporter [34]. In contrast, (+)-M1 acts as a potent MOR agonist. The observation that the opioid receptor competitive antagonist naloxone only partially antagonizes the analgesic effects of tramadol supports the conclusion that its analgesic action is mediated through multiple mechanisms [26].

#### 2.2.3. Key Targets and Signaling Pathways

Opioid receptor activation is the most established mechanism underlying the analgesic effects of tramadol. Tramadol exhibits weak affinity for the MOR and suppresses nociceptive signal transmission in the central nervous system through receptor activation [32]. Monoamine transporters represent an additional major mechanism of action. By inhibiting the reuptake of 5-HT and norepinephrine, tramadol increases synaptic concentrations of these monoamine neurotransmitters in the central nervous system, thereby attenuating pain signal transmission [35]. Serotonin receptors (5-HTRs), which are predominantly members of the G protein-coupled receptor (GPCR) family—with the exception of the 5-HT3 receptor, a ligand-gated ion channel—are involved in diverse physiological processes. Experimental evidence indicates that tramadol exerts antinociceptive effects via serotonergic mechanisms mediated by 5-HT2 receptors [36]. Inhibition of the serotonin transporter (SERT) and norepinephrine transporter (NET) is the primary mechanism responsible for reduced monoamine reuptake [37]. Norepinephrine (NE) effects are mediated by adrenergic receptors (ARs), divided into two main categories, α and β. ARs are GPCRs divided into 9 subtypes encoded by different genes: three α_1_ ARs, three α_2_ ARs and three β_1_ ARs, Tramadol exerts its antinociceptive effect by inhibiting the NET, thereby increasing the concentration of norepinephrine in the synaptic cleft, and indirectly activating downstream α_2_-adrenergic receptors [38].

## 3. Core Modification Strategies and Representative Research Advances

### 3.1. Synergistic Analgesic Strategies

This strategy aims to achieve multitarget synergistic analgesia by integrating tramadol with other analgesic agents that act through distinct mechanisms into a single chemical or physical entity. Such an approach seeks to produce a “1 + 1 > 2” synergistic effect, potentially allowing for dose reductions of individual components and thereby improving overall safety profiles.

#### 3.1.1. Hybrid Molecules and Synergistic Prodrugs

Non-steroidal anti-inflammatory drugs (NSAIDs), such as mefenamic acid, diclofenac sodium, and ibuprofen, have demonstrated therapeutic efficacy in various inflammatory diseases [39]. However, long-term NSAID use is often associated with adverse gastrointestinal effects, including irritation, ulceration, and hemorrhage, which limit clinical utility [40,41]. Hybrid molecules, or synergistic prodrugs, are formed by chemically coupling two drug molecules, in which each active moiety may facilitate the delivery or pharmacological action of the other. The selected components may possess biological activities similar to those of the parent drug, generating synergistic effects, or may confer complementary biological activities absent in the parent compound, resulting in additional therapeutic benefits [42]. In some cases, such hybrids may also function as targeted delivery systems or incorporate components that mitigate adverse effects [43]. Although hybrid molecules are generally multifunctional, the reverse is not necessarily true. Multifunctional ligands may produce multiple pharmacological effects without being true molecular hybrids, for example through a single pharmacophore with pleiotropic activity. In contrast, hybrid compounds are typically defined as chemical entities in which distinct pharmacophoric elements are intentionally integrated into a single molecule through covalent linkage [44,45]. The design of tramadol–NSAID hybrid molecules can be conceptually linked to the principles of the WHO analgesic ladder and multimodal analgesia. Clinical pain management strategies frequently combine weak opioids such as tramadol with NSAIDs to achieve enhanced analgesia through complementary mechanisms acting at both central and peripheral levels. Hybrid molecules integrating tramadol and NSAID pharmacophores translate this therapeutic principle into a single chemical entity, allowing simultaneous modulation of opioid receptors, monoaminergic pain-inhibitory pathways and inflammatory mediators. In this context, such hybrids may be considered a molecular implementation of multimodal analgesia within a unified pharmacokinetic and pharmacodynamic framework [46,47]. Given that combination therapy is widely employed to treat various diseases, often via multiple dosage forms, synergistic prodrug strategies have attracted considerable interest due to their potential to simplify dosing regimens and optimize pharmacodynamic synergy.

In clinical practice, combining tramadol with non-opioid analgesics represents a common therapeutic approach for managing moderate to severe pain [43]. Based on this principle, researchers utilized thionyl chloride to convert various NSAIDs into corresponding acyl chlorides (R–COCl), which were subsequently coupled with tramadol to construct synergistic prodrug systems [43]. This strategy was designed to improve physicochemical properties and enhance the therapeutic activity of NSAIDs by esterifying free carboxyl groups, while simultaneously leveraging the synergistic analgesic effects of NSAIDs and tramadol to achieve more potent and sustained analgesia.

Namdeo et al. [43] synthesized a series of hybrid molecules (synergistic prodrugs) in which tramadol was conjugated with ibuprofen, diclofenac sodium, and mefenamic acid. The structures of these hybrids were characterized by elemental (C, H, N) analysis, infrared spectroscopy, and nuclear magnetic resonance (NMR) spectroscopy. Analgesic activity was evaluated in albino rats using the tail-flick assay. Compounds were administered intraperitoneally at a dose of 50 mg/kg to assess antinociceptive effects. Results indicated that the ibuprofen-based synergistic prodrug exhibited superior analgesic activity compared with prodrugs formed with mefenamic acid and diclofenac sodium. Namdeo et al. also conducted molecular studies using the Schrödinger Suite 2021, in which all hybrid molecules and reference compounds were docked. Results demonstrated that all synthesized synergistic prodrugs exhibited binding affinities to various pain-related receptors, such as KOR (Protein Data Bank, PDB ID 4DJH), DOR (PDB ID 4EJ4) and Cyclooxygenase-2 (COX-2, PDB ID 5F19), which were comparable to those of the corresponding standard monomeric drugs; these docking data were consistent with the observed pharmacological findings. Chemical hydrolysis kinetics were investigated in buffer solutions at 37 °C at pH 1.2 and 7.4. Results showed that, under different pH conditions, the prodrugs were hydrolyzed within 4–6 h to release the parent drugs, which then exert analgesic effects. Nevertheless, the clinical translation potential of these compounds requires comprehensive preclinical safety evaluations and further validation through clinical trials.

#### 3.1.2. Active Pharmaceutical Ingredient–Active Pharmaceutical Ingredient Cocrystals

The US Food and Drug Administration (FDA) defines cocrystals as crystalline materials composed of two or more different molecular entities within the same crystal lattice, held together by nonionic and noncovalent interactions [48]. Active pharmaceutical ingredient–active pharmaceutical ingredient (API–API) cocrystals represent a distinct solid-state form [49] that is fundamentally different from loose combinations or fixed-dose combinations (FDCs). Specifically, API–API cocrystals contain more than one active component within a single crystalline phase rather than being simple physical mixtures of individual drugs [50]. A notable advantage of API–API cocrystals is their ability to enable the simultaneous release of active components, which may lead to unique pharmacodynamic interactions and synergistic effects in clinical settings [51].

The tramadol–celecoxib cocrystal (CTC) is an API–API cocrystal developed for the treatment of acute pain [52] and is the first analgesic cocrystal comprising two active pharmaceutical ingredients. In its crystal structure, racemic tramadol hydrochloride and celecoxib are present in a 1:1 molecular ratio within a supramolecular crystal network [53,54]. Together, these two APIs encompass four major pain-modulating pathways: tramadol mediates MOR agonism and inhibits norepinephrine and 5-HT reuptake (central analgesia), while celecoxib selectively inhibits COX-2, providing peripheral anti-inflammatory and analgesic effects [52]. This multimodal mechanism of action enables the combination to deliver both centrally and peripherally mediated analgesia simultaneously and has the potential to improve both efficacy and safety profiles compared with existing pain therapies [52,55].

The clinical formulation of CTC is an oral immediate-release tablet containing 44 mg of tramadol and 56 mg of celecoxib. CTC 200 mg (administered twice daily) was first approved in the United States in 2021 [56] and subsequently received regulatory approval in Europe in September 2023, with Spain being the first country to market the product, for the treatment of acute moderate-to-severe somatic pain in adults [57]. Compared with tramadol monotherapy, CTC achieves analgesic efficacy at lower daily doses and may exhibit an improved adverse event profile [52]. For acute pain, immediate-release tramadol is typically initiated at 100 mg, followed by 50–100 mg every 4–6 h as needed, with a maximum daily dose of 400 mg [58]. Phase 3 clinical trials in patients with moderate-to-severe acute pain demonstrated that, over a 48 h treatment period, CTC 200 mg administered twice daily was better tolerated than immediate-release tramadol 100 mg administered four times daily, with a lower incidence of opioid-related adverse events [59]. To evaluate tolerability upon first administration (when adverse events and treatment discontinuations are most likely to occur), Morte et al. [60] analyzed safety data from a pivotal Phase 3 trial of CTC in patients with moderate-to-severe acute postoperative pain. They compared the incidence of common opioid-related adverse events following first doses of CTC 200 mg (containing 88 mg tramadol) versus immediate-release tramadol 50 mg. The study results demonstrated that CTC provides superior analgesic efficacy while maintaining similar tolerability to tramadol 50 mg upon first administration. These findings may help clinicians mitigate concerns regarding opioid-related adverse events at the initiation of CTC therapy.

In summary, CTC employs supramolecular crystallization techniques to enable the coordinated delivery of tramadol and celecoxib. By leveraging a multimodal mechanism of action, CTC achieves clinical advantages of lower dosages, high efficacy, and high tolerability, providing an optimized approach for the treatment of acute moderate-to-severe pain.

### 3.2. Mechanism-Balancing and Optimization Strategy

This strategy involves rebalancing the pharmacological profile of tramadol. Structural modifications are employed to substantially enhance MOR agonist activity to improve analgesic efficacy, while maintaining or reinforcing norepinephrine reuptake inhibition (NRI) to strengthen descending inhibitory pathways and simultaneously attenuating serotonin reuptake inhibition. The goal is to mitigate serotonergic adverse effects, such as nausea and vomiting, and reduce reliance on CYP2D6-mediated metabolism.

Tramadol acts as a weak MOR agonist and also exhibits inhibitory activity toward 5-HT and norepinephrine reuptake [61]. However, its analgesic efficacy is largely dependent on the formation of the active metabolite M1 via CYP2D6-mediated metabolism [62], and genetic polymorphisms of this enzyme lead to pronounced interindividual variability in clinical response. Monoaminergic systems play a critical role in descending inhibitory pathways that modulate spinal pain transmission. Accordingly, inhibition of norepinephrine and 5-HT reuptake represents a complementary analgesic mechanism [63]. Further studies have demonstrated that NRI exerts more potent analgesic effects than serotonin reuptake inhibition [64,65]. In tramadol, serotonergic activity contributes only modestly to analgesia but represents a primary source of adverse effects, such as nausea and vomiting. Moreover, because both the analgesic effects and adverse reactions of opioid drugs are mediated by the same receptor subtype, complete separation of efficacy and toxicity is not feasible [66].

Consequently, the mechanism-balancing strategy is based on the concept of enhancing core analgesic pathways while attenuating redundant adverse-effect pathways. This approach aims to generate molecules with combined MOR agonist and NRI activity through chemical modification, thereby exploiting dual-mechanism synergy to enhance analgesic efficacy while reducing the risk of adverse effects associated with excessive activation of a single target [67].

#### 3.2.1. Representative Advances

**Figure 3:**
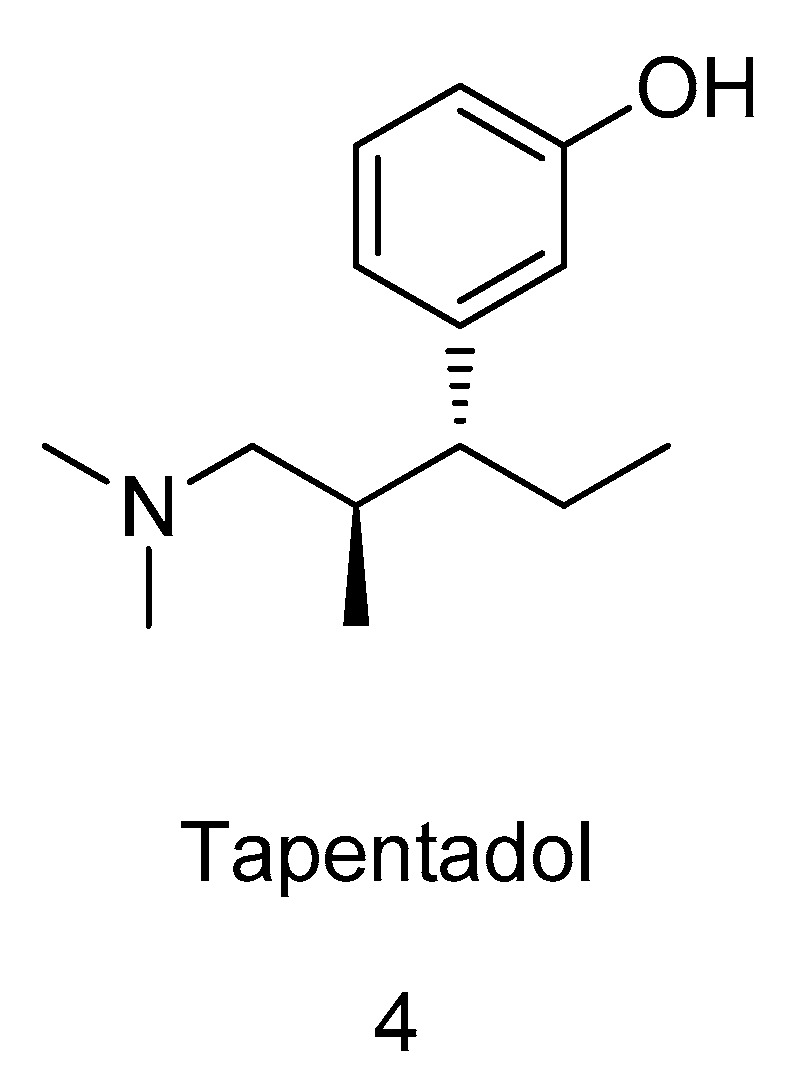
Structure of tapentadol (**4**).

Tapentadol (**4**, Figure 3) represents a successful implementation of this mechanism-balancing strategy. In 2007, Tzschentke et al. [67] evaluated the receptor-binding profile of tapentadol and reported its affinities for rat opioid receptors, expressed as Ki values (the inhibition constant; it reflects the binding affinity between an inhibitor and its target, with a lower Ki value indicating higher ligand–receptor binding affinity): MOR, 0.096 μM; δ-opioid receptor (DOR), 0.97 μM; and κ-opioid receptor (KOR), 0.91 μM. In addition, tapentadol exhibited a Ki value of 0.16 μM for binding to the human recombinant MOR. To assess the effects of tapentadol on monoamine neurotransmitter reuptake, synaptosomes were prepared from neural tissues under isotonic conditions using gentle homogenization, followed by purification via differential centrifugation and density gradient centrifugation. Norepinephrine uptake was measured in rat hypothalamic tissue, while 5-HT uptake was assessed in tissue from the medulla oblongata and pons. The corresponding Ki values were 0.48 μM for NRI and 2.37 μM for serotonin uptake inhibition, indicating higher specificity and selectivity for NRI. No significant activity was observed in binding assays targeting γ-aminobutyric acid (GABA) or dopamine receptors. Notably, the primary metabolite of tapentadol, tapentadol O-glucuronide, lacks analgesic activity. This feature represents an important distinction from tramadol regarding pharmacogenetic variability, as the analgesic efficacy of tramadol depends on an active metabolite whose formation is influenced by CYP2D6 genetic polymorphisms.

Clinical trials have demonstrated that patients with neuropathic pain treated with tapentadol alone or in combination with pregabalin—an anticonvulsant that modulates high-voltage-activated calcium channels—show significant improvements in outcomes and quality-of-life measures [68]. Simultaneously, compared with strong opioids and anticonvulsant agents, tapentadol- or tapentadol/pregabalin-based therapy exhibits favorable tolerability profiles [68]. In addition, in patients with low back pain accompanied by a neuropathic pain component, a treatment regimen of tapentadol extended-release (ER) at a daily dose of 300 mg produced more pronounced clinical improvements [69]. With respect to safety, tapentadol ER shows no significant differences compared with oxycodone/naloxone ER. However, tapentadol ER demonstrates a significant advantage in reducing pain intensity relative to oxycodone/naloxone ER. Compared with oxycodone/naloxone ER, tapentadol ER significantly improves scores on the painDETECT questionnaire and the Neuropathic Pain Symptom Inventory. Both clinical relevance and statistical significance indicate superior efficacy of tapentadol ER. Consequently, for patients with severe chronic low back pain accompanied by a neuropathic pain component, tapentadol ER may be considered a first-line therapeutic option [70].

Through directed structural modification of tramadol, advances in both mechanism of action and pharmacokinetic properties have been achieved, resulting in compounds that function as both MOR agonists and NRIs. The recognition that 5-HT and norepinephrine participate in pain pathways [71] has provided new perspectives on the mechanism of action of tramadol as an atypical opioid analgesic. Tramadol mediates pain control through the integrated involvement of MOR, 5-HT, and norepinephrine signaling pathways. Tapentadol shares mechanistic similarities with tramadol in that it targets both MOR activation and NRI; however, unlike tramadol, tapentadol is not constrained by tramadol’s metabolic characteristics, including dependence on CYP2D6-mediated bioactivation [72].

#### 3.2.2. Comparison of the Pharmacological Characteristics of Tramadol and Tapentadol

Faria [73] compared tramadol and tapentadol with respect to adverse effects, toxicity, advantages, and limitations. Tramadol is a prodrug whose opioid activity is primarily mediated by M1, formed through metabolic activation, and it also inhibits the reuptake of norepinephrine and 5-HT. In contrast, tapentadol does not require metabolic activation and exerts its analgesic effects mainly through NRI combined with potent MOR agonism. Tapentadol exhibits weaker serotonergic activity, more linear pharmacokinetics, improved gastrointestinal tolerability, and greater suitability for the treatment of chronic and neuropathic pain. Nevertheless, both in vitro and in vivo studies indicate that tramadol and tapentadol can induce similar toxicological effects. Therefore, the selection of opioid analgesics should be an individualized, carefully balanced, and patient-tailored decision, taking into account prior treatment experience, patient-specific factors, pain etiology, and the overall therapeutic context.

Roulet et al. [4] further demonstrated that both tramadol and tapentadol combine MOR agonism with monoaminergic activity. However, the analgesic potency of tapentadol is approximately 2- to 3-fold greater than that of tramadol and roughly 1/3 to 1/2 that of morphine. Tapentadol does not produce metabolites with intrinsic analgesic activity and does not rely on CYP-mediated metabolism, thereby avoiding the drug–drug interaction risks associated with tramadol as well as interindividual variability resulting from CYP enzyme genetic polymorphisms. Although the toxicological profiles of the two agents are broadly similar, tapentadol is associated with fewer serotonergic adverse effects (such as nausea, vomiting, and hypoglycemia) but a higher incidence of typical opioid-related adverse effects, including constipation, respiratory depression, and abuse potential.

In summary, as a representative agent of the mechanism-balancing strategy, tapentadol achieves the advantages of potent analgesia, rapid onset of action, and reduced interindividual variability. Nevertheless, it is not generally considered a first-line opioid analgesic [4], but rather a complementary option within pain management. After careful evaluation of individual patient characteristics, comorbidities, and concomitant medications, certain patients may derive clinical benefit from tapentadol therapy.

Tramadol itself is a prototypical dual-mechanism analgesic, possessing both weak μ-opioid receptor agonism and norepinephrine/serotonin reuptake inhibition activities. This pharmacological profile has provided an important concept for the development of multifunctional opioid ligands, namely integrating opioid receptor modulation with other analgesia-related mechanisms within a single molecule. Representative examples include tapentadol, which exhibits both μ-opioid receptor agonism and norepinephrine reuptake inhibition (MOR-NRI) activities [74], and cebranopadol, which acts on both the nociceptin/orphanin FQ peptide (NOP) receptor and classical opioid receptors [75]. Overall, both direct structural modification based on the tramadol scaffold and extension of its “dual-mechanism” pharmacological model suggest that the design of tramadol-related hybrids or dual-mechanism analgesics is a promising research and development pathway. However, not all of these compounds are strictly tramadol-derived hybrids in a chemical sense; however, they are representative examples of multifunctional opioid ligands conceptually related to tramadol’s dual-mechanism analgesic profile.

### 3.3. Metabolic Optimization Strategy

The analgesic efficacy of tramadol in vivo largely depends on its active metabolite M1 (O-desmethyltramadol, **3**), which is generated via metabolism by CYP2D6 [1]. However, CYP2D6 exhibits substantial genetic polymorphism, resulting in marked interindividual differences in metabolic rate, drug accumulation, and clearance, which in turn affect both therapeutic efficacy and safety. Based on CYP2D6 activity, patients can be classified as poor metabolizers (PMs), intermediate metabolizers (IMs), extensive (normal) metabolizers (EMs), and ultrarapid metabolizers (UMs) [12]. In patients with a PM phenotype [4], reduced CYP2D6 activity leads to higher plasma concentrations of the parent drug tramadol and lower levels of M1, resulting in diminished opioid-mediated analgesic efficacy. In this population, plasma tramadol concentrations are approximately 20% higher than those observed in normal metabolizers, whereas M1 concentrations are reduced by approximately 40% [76]. Conversely, in patients with a UM phenotype [4], elevated CYP2D6 activity results in rapid tramadol metabolism, often necessitating higher doses to achieve analgesic efficacy. This can lead to increased formation of M1, thereby enhancing opioid effects and toxicity [77]. It is estimated that approximately 30–50% of patients experience an increased risk of adverse effects due to such metabolic variability. Overall, the metabolic pathways and pharmacokinetic characteristics of tramadol are strongly influenced by CYP2D6 genetic polymorphisms, which can substantially compromise efficacy and safety. Consequently, the administration of M1 provides a means to circumvent these risks.

Therefore, direct administration of M1 can bypass the metabolic bottleneck and avoid interference from CYP2D6 genetic polymorphisms. In June 2019, Syntrix Pharmaceuticals conducted two consecutive double-blind, randomized, placebo-controlled, three-period crossover studies (Study A and Study B) to evaluate the steady-state pharmacokinetics and analgesic effects of 20 mg M1 and 50 mg tramadol in 103 healthy participants. Among these participants, 43 did not receive the inhibitor paroxetine (paroxetine is a selective serotonin reuptake inhibitor (SSRI) used for the treatment of depression and neuropathic pain; it is primarily metabolized by CYP2D6, and genetic polymorphisms or inhibition of CYP2D6 can significantly affect its metabolic activity and plasma concentration), whereas 60 received concomitant paroxetine treatment. Under conditions without CYP inhibition (Study A), administration of 20 mg M1 or 50 mg tramadol every 6 h produced comparable steady-state plasma levels of (+)-M1, similar adverse event profiles, and analgesic effects that were significantly superior to placebo and equivalent between the two treatments. In Study B, CYP inhibition markedly reduced steady-state (+)-M1 levels following tramadol administration, decreased tramadol-associated adverse effects, and resulted in analgesic efficacy that was not significantly different from placebo. In contrast, CYP inhibition had no significant effect on exposure to either (+)-M1 or (−)-M1 following M1 administration, and its analgesic efficacy remained comparable to that observed in Study A and was superior to that of tramadol (*p* = 0.003). These results indicate that M1 retains the safety and efficacy profile of tramadol while circumventing metabolic limitations [34]. In 2021, the company further investigated the dose proportionality and food effects of orally administered M1 in healthy subjects, aiming to determine systemic exposure to M1 at different oral dose levels (https://clinicaltrials.gov/study/NCT04683926?cond=desmetramadol&rank=1#more-information, accessed on 13 October 2025).

**Figure 4:**
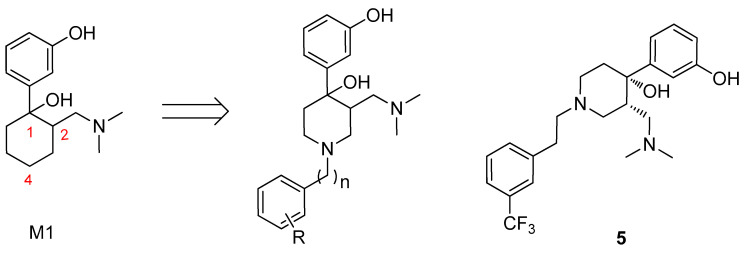
Compound design basis of M1 and structure of Compound **5**.

Huang et al. [78] used M1 as a starting point to design and synthesize a series of 3-((dimethylamino)methyl)-4-(3-hydroxyphenyl)-1-phenethylpiperidin-4-ol analogues and evaluated their in vitro activities. Based on M1, they sought to develop bifunctional ligands exhibiting MOR agonism and DOR antagonism through structural modification at the 4-position. To simplify the synthetic route and reduce chiral complexity, the carbon atom at the 4-position was replaced with a nitrogen atom, which was subsequently linked to a phenyl group. The results indicated that Compound **5** was the most selective and potent MOR agonist in the series, as shown in Figure 4. Structure–activity relationship studies demonstrated that both the linker between the piperidine ring and the substituted phenyl ring and the substitution pattern on the phenyl ring were critical determinants of opioid activity and receptor selectivity. This compound was thus identified as a selective MOR agonist based on a novel molecular scaffold.

Although this strategy directly addresses the fundamental metabolic limitations of tramadol, even highly potent candidate compounds derived in this manner remain subject to the inherent risks associated with opioid pharmacology and must undergo comprehensive preclinical and clinical development to establish overall therapeutic value.

### 3.4. Pharmacophore Optimization Strategy

This strategy is based on an in-depth understanding of the microscopic binding modes of tramadol and morphine at the MOR. By chemically modifying the core structures of tramadol and M1, this approach is designed to explore novel ligand–receptor interaction patterns and to identify derivatives with potent activity, receptor subtype selectivity, and novel pharmacological properties.

#### 3.4.1. Pharmacophore Homology Between M1 and Morphine

In recent years, tramadol has frequently been regarded as structurally related to codeine [23,79,80]. Three-dimensional (3D) superposition analyses of tramadol and codeine have revealed that the nitrogen atom and the 3-methoxyphenyl moiety of both compounds occupy similar spatial positions, suggesting that tramadol and codeine share common pharmacophoric features. Given that tramadol exerts its *µ*-opioid activity primarily through its O-demethylated metabolite M1, and that morphine is likewise the more active O-demethylated metabolite of codeine, 3D superposition analyses were also performed for M1 and morphine. These analyses showed that the two compounds possess highly similar pharmacophoric characteristics [24]. Molecular docking of M1 and morphine into the crystal structure of the MOR further demonstrated that the protonated nitrogen atoms of both ligands form a salt bridge with Asp147^3.32^ (the aspartic acid residue at position 32 of transmembrane helix 3, corresponding to residue 147 in the receptor sequence), while their phenolic hydroxyl groups participate in a hydrogen-bonding network mediated by water molecules. These binding interactions are consistent with the canonical binding mode of morphinan compounds observed in MOR crystal structures [81,82].

Combined 3D structural superposition and molecular docking studies have confirmed that M1 and morphine share identical pharmacophoric features and highly similar binding modes at the MOR. This provides a theoretical framework for derivative design: by retaining the core pharmacophores homologous to those of morphine—namely the protonated nitrogen atom, the phenolic hydroxyl group, and the 3-substituted phenyl ring—structural modifications of the M1 scaffold can preserve specific opioid receptor binding while allowing the introduction of additional substituents to establish novel interactions, thereby optimizing potency and receptor selectivity [24].

#### 3.4.2. Representative Research Advances

**Figure 5:**
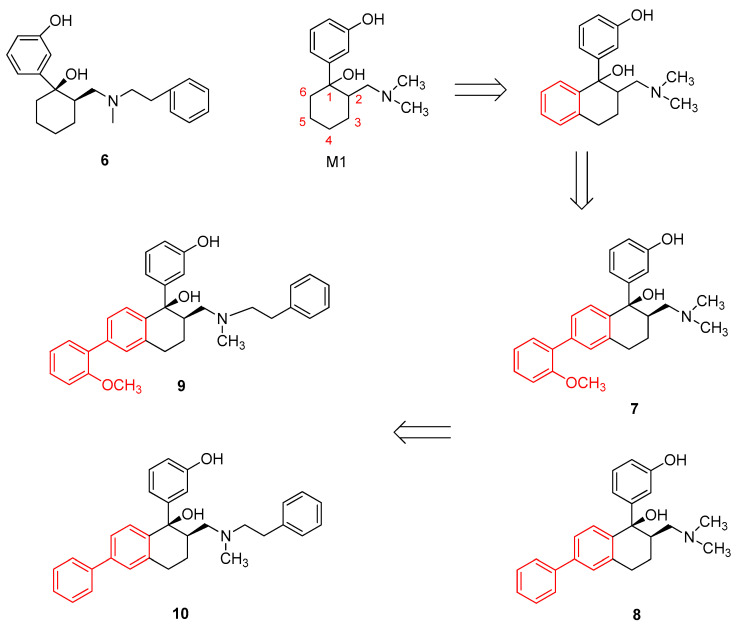
Structures of Compound **6**, Compound **7**, Compound **8**, Compound **9** and Compound **10**.

Shen et al. [24] employed 3D structural superposition and molecular docking to demonstrate that M1 and morphine share common pharmacophoric features and exhibit similar binding modes at the MOR. Following successful strategies from morphine structural modification—specifically introducing an N-phenethyl substituent to enhance opioid activity through hydrophobic interactions with Trp293^6.48^ and Tyr326^7.43^—the researchers designed, synthesized, and evaluated a series of N-phenylalkyl-substituted tramadol derivatives for opioid activity. Among them, the N-phenethyl-substituted compound (Compound **6**, Figure 5) displayed functional activity comparable to that of M1. Further molecular docking studies revealed that, unlike N-phenethyl morphine, this compound adopted a distinct binding orientation: its N-phenethyl moiety extended into an alternative hydrophobic pocket within the second extracellular loop (ECL2), formed by residues Ile144^3.29^, Val143^3.28^, and Leu219. These findings suggest that introducing an additional hydrogen-bond donor at the phenolic hydroxyl group could potentially re-establish the original water-mediated hydrogen-bonding network, thereby enhancing interactions with the MOR and improving activity. Additionally, occupation of this hydrophobic pocket by the N-phenethyl side chain may enhance hydrophobic contacts and help stabilize the ligand–receptor complex, thereby contributing to the observed opioid activity.

The classical message−address concept [83] was originally proposed to characterize the binding mechanisms between opioid receptors and their endogenous peptide ligands (e.g., DOR and enkephalins). The N-terminal residues of the peptide are defined as the message domain, which is responsible for receptor recognition and binding affinity, whereas residues in the central or C-terminal regions constitute the address domain, conferring receptor subtype selectivity or enhanced efficacy. This concept was subsequently extended to the discovery of the first nonpeptidic DOR antagonists and agonists, such as naltrindole (NTI) and 7-spiroindanyloxymorphone (SIOM) [84,85]. In these compounds, the morphinan scaffold was designed to serve as the message, while the appended aromatic moieties (e.g., the indole group in NTI) functioned as the address responsible for DOR selectivity.

Shen et al. [86] applied this concept to the design of tramadol derivatives. By performing 3D structural superposition of (1*R*,2*R*)-M1 with SIOM and morphine, they found that the nitrogen atom and phenolic moiety of the former two compounds were highly congruent, indicating that (1*R*,2*R*)-M1 shares pharmacophoric features with both morphine and SIOM. According to the message−address concept, the nitrogen atom and phenolic fragment of these compounds can be regarded as the message domain responsible for DOR activity. Inspired by the identification of NTI and SIOM, they introduced an address element into the desmethyltramadol scaffold by appending a biphenyl group to the cyclohexyl ring and subsequently synthesized a series of derivatives. They developed aminomethyl tetrahydronaphthalene derivatives, leading to the identification of a potent DOR agonist, Compound **7**, and a moderately active KOR agonist, Compound **8** (Figure 5). Compound **7** was identified as a key finding. Molecular docking studies indicated that the message module of Compound **7** activates the receptor via a salt bridge with Asp128^3.32^ and hydrogen-bond interactions with adjacent water molecules, while its “address” module engages in cation–π interactions with Lys214^5.39^ and spatial packing with hydrophobic residues including Leu300^7.35^, Val281^6.55^, and Trp284^6.58^. Notably, the specific interaction with Leu300^7.35^ constitutes the key structural basis for the DOR selectivity of Compound **7**. Compound **7** may serve as a lead molecule to guide further pharmacological investigations. Mechanistically, the biphenyl “address” moiety may occupy a relatively hydrophobic subpocket within the DOR binding site and establish additional hydrophobic and π interactions, thereby stabilizing the ligand–receptor complex and enhancing its DOR selectivity. Given their notable pharmacological profiles, the aminomethyl tetrahydronaphthalene scaffold was selected as a research template to provide insights into the molecular mechanisms governing the interactions of tramadol and structurally related analogs with different opioid receptor subtypes. These compounds may serve as molecular probes to investigate the structural determinants of receptor subtype selectivity and the mechanisms underlying opioid receptor activation. However, to date, no computational studies have explained the binding modes, selectivity, or activation mechanisms of these analogs. To address this gap, Xie et al. [87] designed and synthesized additional aminomethyl tetrahydronaphthalene probes, including Compound **9** and Compound **10** (Figure 5), and evaluated their activity across opioid receptor subtypes. Subsequent molecular docking and molecular dynamics simulations were employed to investigate the structural determinants underlying receptor subtype selectivity and activation mechanisms. GPCRs are integral membrane proteins characterized by seven transmembrane α-helical domains (TM1–TM7) connected by three intracellular and three extracellular loops. The results identified Y^7.43^ (the tyrosine residue at position 43 of transmembrane helix 7, TM7) as a key determinant of subtype selectivity across the three opioid receptors, while W^6.58^ (the tryptophan residue at position 58 of TM6) was critical for DOR selectivity. A stepwise signal transduction mechanism was proposed for aminomethyl tetrahydronaphthalene-induced activation of the three opioid receptors: first, the formation of the TM3–TM7 “3–7 lock”; second, establishment of the TM3–TM6 DRG (Asp–Arg–Gly) lock; and third, the rearrangement of I^3.40^ (isoleucine), P^5.50^ (proline), and F^6.44^ (phenylalanine), which triggers coordinated movements of all seven transmembrane helices (7 TMs). This structural relaxation on the intracellular side creates a binding pocket for G protein coupling, ultimately leading to receptor activation. These findings highlight the significance of the aminomethyl tetrahydronaphthalene scaffold in the design and screening of novel, potent, and selective opioid receptor ligands. Mechanistically, the rigid tetrahydronaphthalene scaffold may help stabilize the spatial orientation of the ligand within the receptor binding pocket and promote receptor conformational changes through stable hydrophobic and aromatic interactions with key transmembrane residues, thereby enhancing receptor subtype selectivity.

## 4. Clinical Translation Challenges and Future Research Prospects for Tramadol Derivatives

### 4.1. Clinical Translation Challenges of Tramadol Derivatives

Tapentadol, as a landmark achievement in tramadol structural modification, has successfully reached the market, validating the developmental potential of this discipline. However, most tramadol derivatives still face multiple bottlenecks in clinical translation:

The challenge of precisely balancing dual mechanisms. The core advantage of tramadol-class drugs lies in the synergistic mechanism of MOR agonism and monoamine reuptake inhibition. This dual activity also constitutes a central obstacle to translation: excessive MOR agonism can exacerbate traditional opioid-related risks, such as respiratory depression and addiction, while overly potent 5-HT reuptake inhibition increases the likelihood of central nervous system adverse events, including serotonin syndrome. Determining the optimal efficacy–safety balance between these two mechanisms remains a key challenge in structural design and activity optimization.

Inherent constraints of opioid-related risks. As long as derivatives retain MOR agonist activity, they cannot completely escape the intrinsic risks associated with opioids. Adverse effects such as respiratory depression, physical dependence, psychological addiction, and constipation remain unavoidable [88]. Although structural modifications may attenuate the severity of these risks, they are difficult to eliminate entirely, constituting a persistent safety barrier to clinical translation.

Pharmacokinetic complexity and interindividual variability. Although derivatives such as tapentadol partially address interindividual variability by circumventing CYP2D6-mediated metabolism, novel tramadol derivatives may involve metabolism mediated by other CYP enzymes or transporters, resulting in continued pharmacokinetic complexity. This necessitates large-scale drug–drug interaction studies, and dose optimization in patients with hepatic or renal impairment, as well as in specific genetic subpopulations, remains challenging, further prolonging development timelines.

Commercial and market-related constraints. Against the backdrop of increasingly stringent regulation of opioid analgesics and rising public health concerns, pharmaceutical companies have adopted a cautious stance toward research and development in this area. The development of novel analgesics is associated with high costs and prolonged clinical timelines, requiring long-term follow-up to assess safety and abuse potential, while the uncertainty of market returns further discourages investment. Consequently, many promising candidates are discontinued at early stages of development.

Technical barriers in clinical trial design. The subjective nature of pain makes efficacy endpoints difficult to quantify precisely and necessitates rigorously designed controlled trials, often requiring comparisons with NSAIDs, acetaminophen, or placebo. For chronic noncancer pain, long-term follow-up is essential to evaluate sustained safety and addiction risk. These requirements not only increase the complexity of trial design but also substantially increase development costs.

### 4.2. Future Research Directions and Outlook

Structural modification of tramadol has evolved from simple functional group alterations to rational drug design grounded in mechanistic understanding and target structural insights. Future research in this field is expected to progress toward greater precision and diversification.

First, precise targeting and mechanism optimization will remain the core focus. Future studies will move beyond the straightforward combination of dual mechanisms and instead focus on fine-tuning the relative contributions of distinct signaling pathways. For example, further refinement of mechanism rebalancing strategies exemplified by tapentadol may yield new molecules with an improved ratio of MOR agonism to NRI, thereby achieving potent analgesia while minimizing serotonergic adverse effects and the risk of respiratory depression. In parallel, selective agonism or antagonism of other opioid receptor subtypes, such as DOR and KOR, offers promising avenues for the development of analgesics with reduced addictive potential or additional antidepressant- and anxiolytic-like properties. Compounds identified using the message–address concept, such as Compound **10**, exhibit significant potential for further exploration [86].

Second, the integration of emerging technology platforms is expected to accelerate lead compound discovery. Artificial intelligence and machine learning will play a prominent role in structure–activity relationship analyses, enabling the mining of large-scale pharmacological datasets to predict the activity, selectivity, and pharmacokinetic properties of novel derivatives, thereby substantially improving research and development efficiency.

Third, innovation in drug delivery systems and combination therapies represents a key driver of clinical translation. The success of CTC has demonstrated the clinical value of integrating multimodal analgesic mechanisms within a single entity. In the future, additional API-API cocrystals or FDC formulations are likely to be explored to achieve synergistic efficacy while reducing the required doses of individual components. Moreover, for specific scenarios such as postoperative pain, the development of local delivery systems (e.g., transdermal patches, locally injectable sustained-release microspheres) offers unique advantages: in addition to μ-opioid receptor activation and monoamine neurotransmitter reuptake inhibition already mentioned in this article, tramadol possesses a non-opioid receptor-mediated local anesthetic-like effect, characterized by concentration-dependent and state-selective inhibition of voltage-gated sodium channels, as well as mild and specific blockage of sensory nerve conduction [89], and this nerve inhibitory effect cannot be mimicked by its major metabolite M1 [90]. Local delivery systems enable the enrichment of tramadol at the target site, leveraging its multimodal synergistic analgesia while minimizing systemic exposure, thereby fundamentally improving safety.

Finally, addressing the opioid-related public health crisis is an essential responsibility in future analgesic development. The development of any novel tramadol derivative must prioritize low abuse and addiction potential as a core evaluation criterion. This requires rigorous preclinical and clinical investigations to systematically assess both physical dependence and psychological addiction liability. In parallel, strengthening research on personalized pharmacotherapy by integrating patient-specific factors such as genetic polymorphisms (e.g., CYP enzymes), comorbidities, and concomitant medications will be essential to provide precise dosing and treatment guidance. This ensures that therapeutic efficacy is achieved while minimizing risks.

In summary, despite the inherent constraints imposed by opioid-associated risks, the prospects remain promising for the development of next-generation analgesics with improved targeting, safety, and efficacy by leveraging tramadol as a structural starting point, supported by multidisciplinary integration and continuous technological innovation. Future success will favor rationally designed molecules capable of achieving an optimal balance among potent analgesia, multimodal mechanisms of action, and superior safety profiles.

## 5. Conclusions

The structural modification of tramadol has evolved from straightforward chemical alterations to mechanism- and structure-based rational drug design. This progression has led to important achievements, including tapentadol and CTC. Although significant challenges remain, particularly those associated with the intrinsic risks of opioid pharmacology, ongoing advances in pain biology, receptor pharmacology, and emerging technologies continue to expand opportunities in this discipline. Consequently, the development of next-generation analgesics that are more targeted, safer, and more effective, using tramadol as a foundational scaffold, remains a realistic and promising objective.

## Data Availability

No new data were created or analyzed in this study. Data sharing is not applicable to this article.

## Abbreviations

API	Active pharmaceutical ingredient
COX-2	Cyclooxygenase-2
CRP	C-reactive protein
CTC	Tramadol–celecoxib cocrystal
CYP	Cytochrome P450
DOR	δ-opioid receptor
ECL2	Second extracellular loop
EM	Extensive (normal) metabolizer
ER	Extended-release
FDCs	Fixed-dose combinations
GPCR	G protein-coupled receptor
IL	Interleukin (e.g., IL-2, IL-6, IL-8)
IM	Intermediate metabolizer
KOR	κ-opioid receptor
LPS	Lipopolysaccharide
M1	O-desmethyltramadol (primary active metabolite)
MOR	μ-opioid receptor
NET	Norepinephrine transporter
NK	Natural killer (cells)
NMR	Nuclear magnetic resonance
NRI	Norepinephrine reuptake inhibition
NSAIDs	Nonsteroidal anti-inflammatory drugs
PM	Poor metabolizer
5-HT	Serotonin (5-hydroxytryptamine)
SAR	Structure–activity relationship
SERT	Serotonin transporter
SIOM	7-spiroindanyloxymorphone
TM	Transmembrane (helix)
TNF	Tumor necrosis factor
UM	Ultrarapid metabolizer
